# Complete plastome sequence of *Lilium pardalinum* Kellogg (Liliaceae)

**DOI:** 10.1080/23802359.2018.1463826

**Published:** 2018-04-23

**Authors:** Hyoung Tae Kim, Peter J. Zale, Ki-Byung Lim

**Affiliations:** a Department of Horticulture, Kyungpook National University, Daegu, Republic of Korea;; b Institute of Agricultural Science and Technology, Kyungpook National University, Daegu, Republic of Korea;; c Longwood Gardens, Kennett Square, PA, USA

**Keywords:** *Lilium pardalinum*, plastome, phylogeny

## Abstract

*Lilium pardalinum* Kellogg is native to the Pacific Coast of the United States, and grows in woodland near streams. In the present study, the complete plastome of *L. pardalinum* was sequenced. The plastome sequence is 151,969 bp long with a large single copy, a small single copy, and two inverted repeat regions of length 81,401, 17,346, and 26,611 bp, respectively. A total of 133 genes were identified, including 82 coding genes, 8 ribosomal RNAs, 38 transfer RNAs, and 5 pseudogenes. Among the 5 pseudogenes, pseudo *ndhF* and *ndhG* genes were similar to that of *L. washingtonianum* and *L. philadelphicum*, respectively. *Lilium pardalinum* is sister to American lilies, except *L. philadelphicum*.

The section *Pseudolirium* Endlicher of the genus *Lilium* consists of *ca.* 20 species inhabiting North America. This section was considered to be very closely related to section Martagon because of whorled leaves (Comber 1949; Lighty 1968). However, the most recent phylogenetic analyses using molecular markers indicated that both the sections are distantly related (Hayashi and Kawano 2000; Lee et al. 2011; Gao et al. 2013; Du et al. 2014). *Lilium pardalinum* Kellogg belongs to the section *Pseudolirium* and is native to the Pacific Coast of the United States, growing in woodland near streams (McRae et al. 1998). In contrast to certain species in the same section, *L. pardalinum* has been successfully used for intersectional hybridization of the genus *Lilium* (van Tuyl and Arens 2010). However, sectional delimitation based on morphological and geographical characters of the genus *Lilium* has been controversial because the recent phylogenetic studies have not strongly supported the sectional classification of the genus *Lilium* (Hayashi and Kawano 2000; Du et al. 2014). Therefore, the genetic studies of *Lilium* species are necessary for systematics and prospective breeding of the genus *Lilium*. In the present study, the plastome of *L. pardalinum* was completely sequenced. The total genomic DNA was extracted from the fresh leaves of *L. pardalinum* using the DNeasy Plant Mini Kit (Qiagen, Valencia, CA, USA). Seeds of *L. pardalinum* were collected at Onion Meadow, Tulare County, California on 28 September 1989 and grew in Kyungpook University, Korea. The living material and DNA were stored in Kyungpook University, Korea. The total DNA was sequenced using HiSeq 2500 instrument (Illumina, San Diego, CA, USA). All genes were annotated using Geneious (Kearse et al. 2012) by comparing with the previously reported plastome sequences of *Lilium* species. The phylogenetic analysis was performed with the concatenated 78 genes using randomized axelerated maximum likelihood (RAxML) (Stamatakis 2014). The plastome sequences of 22 *Lilium* and 3 *Fritillaria* species were downloaded from the National Center for Biotechnology Information database.

The plastome sequence is 151,969 bp long and consists of a large single copy region (81,401 bp), a small single copy region (17,346 bp), and two inverted repeat regions (26,611 bp). A total of 133 genes were identified, including 82 coding genes, 8 ribosomal RNAs, 38 transfer RNAs, and 5 pseudogenes.

The phylogenetic analysis showed that *L. pardalinum* and *L. washingtonianum* formed a clade, and they were sister to *L. superbum* of the section Pseudolirium (Figure 1). However, these three species were distantly related to *L. philadelphicum*. This polyphyly of the section Pseudolirium has been reported by previous studies (Hayashi and Kawano 2000; Lee et al. 2011); however, certain studies have supported that the section Pseudolirium forms a clade (Nishikawa et al. 2001; Du et al. 2014). However, our result does not imply that *L. philadelphicum* should be distinguished from other Pseudolirium species because there are still taxonomic gaps and questions on the misidentification of samples. Consequently, more phylogenetic studies will help us understand the relationship among the species of the genus *Lilium*.

**Figure 1.**
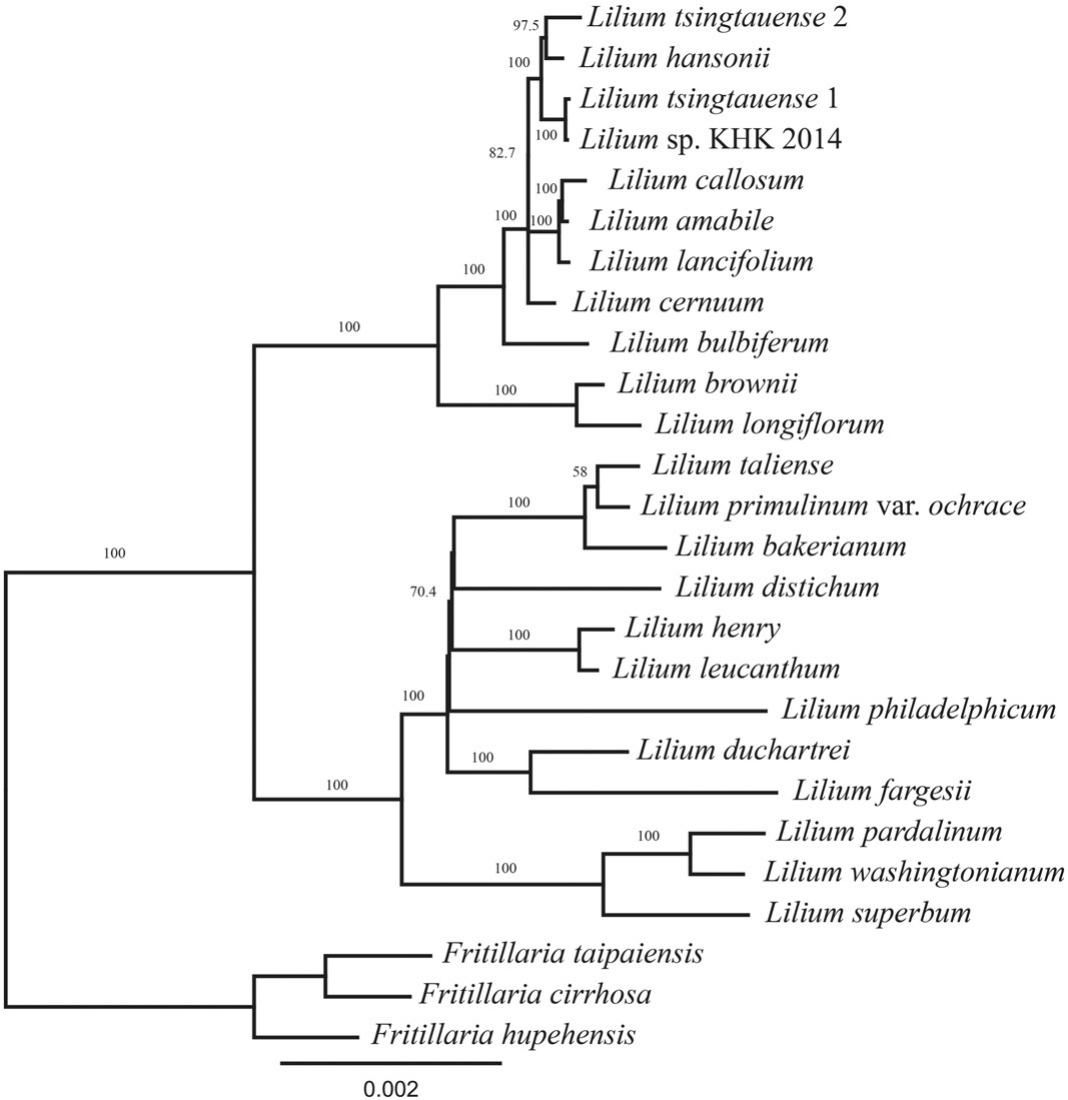
Phylogenetic analysis with 78 genes using randomized axelerated maximum likelihood (RAxML). *Lilium pardalinum* and *L. washingtonianum* form a clade, and they are sister to *L. superbum* of the section *Pseudolirium*. However, *L. philadelphicum*, another species of the section *Pseudolirium*, is distant from rest of the section *Pseudolirium*. The numbers on the node and scale refer to bootstrap values and substitutions per site, respectively.

## References

[CIT0001] Comber HF. 1949 A new classification of the genus *Lilium* Vol. 13 Lily Year Book, Royal Horticultural Society.13:86–105.

[CIT0002] Du YP, He HB, Wang ZX, Li S, Wei C, Yuan XN, Cui Q, Jia GX. 2014 Molecular phylogeny and genetic variation in the genus Lilium native to China based on the internal transcribed spacer sequences of nuclear ribosomal DNA. J Plant Res. 127:249–263. 24212402 10.1007/s10265-013-0600-4

[CIT0003] Gao YD, Harris AJ, Zhou SD, He XJ. 2013 Evolutionary events in *Lilium* (including Nomocharis, Liliaceae) are temporally correlated with orogenies of the Q-T plateau and the Hengduan Mountains. Mol Phylogenet Evol. 68:443–460. 23665039 10.1016/j.ympev.2013.04.026

[CIT0004] Hayashi K, Kawano S. 2000 Molecular systematics of *Lilium* and allied genera (Liliaceae): phylogenetic relationships among *Lilium* and related genera based on the rbcL and matK gene sequence data. Plant Species Biol. 15:73–93.

[CIT0005] Kearse M, Moir R, Wilson A, Stones-Havas S, Cheung M, Sturrock S, Buxton S, Cooper A, Markowitz S, Duran C, et al 2012 Geneious Basic: an integrated and extendable desktop software platform for the organization and analysis of sequence data. Bioinformatics. 28:1647–1649. 22543367 10.1093/bioinformatics/bts199 PMC3371832

[CIT0006] Lee CS, Kim SC, Yeau SH, Lee NS. 2011 Major lineages of the genus *Lilium* (Liliaceae) based on nrDNA ITS sequences, with special emphasis on the Korean species. J Plant Biol. 54:159–171.

[CIT0007] Lighty R. 1968 Evolutionary trends in lilies. Lily Year Book RHS; 31:40–44.

[CIT0009] McRae EA, Austin-Mcrae E, MacRae E. 1998 Lilies: a guide for growers and collectors. Vol. 105 Portland: Timber Press.

[CIT0010] Nishikawa T, Okazaki K, Arakawa K, Nagamine T. 2001 Phylogenetic analysis of section Sinomartagon in genus *Lilium* using sequences of the internal transcribed spacer region in nuclear ribosomal DNA. Breed Sci. 51:39–46.

[CIT0011] Stamatakis A. 2014 RAxML version 8: a tool for phylogenetic analysis and post-analysis of large phylogenies. Bioinformatics. 30:1312–1313. 24451623 10.1093/bioinformatics/btu033 PMC3998144

[CIT0012] van Tuyl JM, Arens P 2011 Lilium: Breeding history of the modern cultiva assortment. Acta Hort. 900:223–230.

